# The Inflation Illusion: How New Zealand Households Overestimate Increases in Food Prices

**DOI:** 10.1002/snz2.70018

**Published:** 2026-02-15

**Authors:** Puneet Vatsa, Alan Renwick

**Affiliations:** ^1^ School of Business and Creative Industries University of the Sunshine Coast Sippy Downs Australia; ^2^ Faculty of Agribusiness and Commerce Lincoln University Lincoln New Zealand

**Keywords:** food price inflation, inflation perceptions, New Zealand, quantile regressions

## Abstract

Using nationally representative survey data collected in February 2025, this article examines how New Zealand households perceive food price inflation. We find that perceived food price inflation significantly exceeds the official rate, with respondents estimating food price inflation to be over seven times higher than reported figures. Perceptions vary by age and gender: women report higher inflation estimates than men, and perceived inflation decreases with age. Quantile regressions suggest that these differences are concentrated around the median and the upper quantile of the distribution of inflation perceptions. These findings highlight a misalignment between statistical reporting and lived experience, with implications for nutrition, wellbeing, and communication of economic data.

## 1 Introduction

Following the end of the food price crisis, which led to high and volatile food prices between 2008 and 2011, New Zealand and many advanced economies experienced an extended period of relatively stable food prices. However, this changed in 2020 with the emergence of the COVID‐19 pandemic, followed closely by a series of climatic and geopolitical shocks. The resultant disruption to the global food supply contributed to high food price inflation. High food prices have attracted considerable attention globally and in New Zealand (Adjemian et al. 2024; FAO, IFAD, UNICEF, WFP, WHO 2025; Vatsa and Renwick 2025). This attention reflects the central role of food in society, as food price inflation has significant implications for food insecurity, poverty, diet, and health (FAO, IFAD, UNICEF, WFP, WHO 2025; Vatsa and Renwick 2025). High inflation also amplifies tensions within the food system and broader society around both its perceived causes (e.g., lack of market competition, export focus of production) and consequences (in terms of fairness and inequality).

Food prices, which account for approximately 10% of the Consumer Price Index (CPI) in New Zealand, exert a substantial influence on the overall inflation rate. Through this channel, they indirectly shape key macroeconomic decisions, including the setting of the Official Cash Rate by the Reserve Bank. While actual price movements are critical, the importance of inflation expectations and perceptions should not be understated. The former is a forward‐looking measure, describing what people think inflation will be in the future. The latter pertains to the past, i.e., what people believe inflation to have been in the past. This distinction is important to make, especially when inflation perceptions diverge from actual inflation and, at the same time, influence inflation expectations (Vatsa and Pino 2025b).

Perceptions influence household and business behaviour, which, in turn, can affect both realised inflation and future inflation expectations (Jonung 1981; Vatsa and Pino 2025b). Although the CPI provides regular and objective measures of food price inflation, it offers limited insight into how inflation is perceived by consumers and how those perceptions shape spending and consumption choices.

Inflation perceptions are influenced by prices that individuals encounter frequently, consistent with the availability heuristic (Tversky and Kahneman 1974), whereby easily recalled or salient experiences disproportionately influence judgements and beliefs. Unsurprisingly, a considerable body of research devoted to the psychology of inflation has analysed food and petrol prices (Binder 2018; Binder and Makridis 2022; Coibion and Gorodnichenko 2015; Kikuchi and Nakazono 2023; Kilian and Zhou 2022a, 2022b, 2025; Vatsa and Pino 2024). The focus of these studies has largely been on inflation expectations, not inflation perceptions. However, Anesti et al. (2025) examined the influence of food prices on both inflation perceptions and expectations in the United Kingdom, and Weber et al. (2022) found a strong correlation between perceived and expected inflation rates in the United States. Furthermore, Anesti et al. (2025) examined differences in experienced inflation across expenditure categories and demographic groups.

Despite these insights, a notable gap remains in our understanding of inflation perceptions within New Zealand, although a few recent studies have begun to engage with this topic (Vatsa 2025; Vatsa and Pino 2025a, 2025b). However, as these studies are based on aggregated data, it is unclear whether such perceptions vary systematically across demographic groups, whether particular food categories (e.g., dairy, meat, vegetables) disproportionately shape these perceptions, and to what extent perceived inflation influences actual purchasing behaviour. Furthermore, although official measures such as food price indices are useful benchmarks for understanding food price trends, they fall short of capturing how households actually experience and respond to rising food costs.

To address this gap, we use novel survey data collected in February 2025 from a representative sample of the New Zealand population to quantify perceptions of food price inflation. We analyse how these perceptions differed across socio‐demographic groups and identify which food categories were most frequently observed. The key findings are these. First, perceptions of food price inflation were consistently higher than official figures across the population, indicating a widespread misalignment between lived experience and statistical reporting by official bodies. Second, age and gender emerged as significant factors, with women reporting higher estimates and perceptions declining with age. Third, evidence on the link between food price inflation and ethnicity is mixed. Furthermore, we find evidence suggesting that food prices are not just shaping how people think about inflation but may also influence what they eat and how much they spend on food.

The remainder of the article is structured as follows. The next section describes the data used in this study. The methods are described in Section 3. Section 4 comprises a discussion of the empirical results, and Section 5 concludes the article.

## 2 Data

We used data from a nationally representative online survey of New Zealand residents conducted from February 3, 2025, to February 14, 2025. The survey comprised questions about demographic characteristics, purchasing habits, perceptions of inflation, and behavioural responses to recent price changes. Respondents were drawn from a commercial panel, comprising over 100,000 active participants, managed by ConsumerLink, a New Zealand‐based firm. The sample was selected to reflect the regional, age, ethnic, and gender distribution of the national population. The respondents were invited to participate in the survey via email.

To elicit perceptions of food price inflation, we presented the respondents with the following question:

By what percentage have food prices changed over the last 12 months? Please provide your own estimate—there are no right or wrong answers, so no need to research or look anything up. Enter a percentage rounded to one decimal place.

Table 1 presents the descriptive statistics of the variables used in this study and highlights a stark discrepancy between actual and perceived food price inflation that emerged from the responses. On average, respondents reported that food prices had risen by approximately 18.14% over the 12 months preceding February 2025. In contrast, official data from Statistics New Zealand put the increase at just 2.90% over the same period.

**TABLE 1:** Descriptive statistics.

Continuous or count variables	Mean	SD
Perceived food price inflation	18.14	19.53
Age	47.52	18.26
Frequency of monthly grocery purchases	5.91	4.67
**Categorical variables**	**Count**	**% of total**
Ethnicity		
Māori	48	6.00%
NZ European + Māori	118	14.75%
NZ European	434	54.25%
Other	200	25.00%
Location		
City centre	63	7.88%
City suburb	469	58.63%
Town	170	21.25%
Village	29	3.62%
Rural	69	8.62%
Gender		
Male	385	48.12%
Female	413	51.62%
Other	2	0.25%
Income (NZD)		
$0–24,999	48	6.00%
$25,000–49,999	128	16.00%
$50,000–74,999	134	16.75%
$75,000–99,999	117	14.62%
$100,000–$124,999	69	8.62%
$125,000–$149,999	47	5.88%
$150,000–$174,999	48	6.00%
$175,000–$199,999	38	4.75%
$200,000 and above	80	10.00%
Undisclosed	91	11.38%
Education		
No or low education	119	14.88%
High‐school diploma	176	22.00%
Vocational or technical diploma	178	22.25%
Bachelor's degree	240	30.00%
Master's degree or above	87	10.88%
Inflation knowledge		
Little or no knowledge	35	4.38%
Limited knowledge	201	25.12%
Moderate knowledge	349	43.62%
Good knowledge	193	24.12%
Excellent knowledge	22	2.75%
Made financial decisions		
Yes	772	96.50%
No	28	3.50%

The average age of the respondents was 47.52 years, and the gender distribution was balanced, with 51.62% identifying as female and 48.12% as male. Just over half of the respondents identified as New Zealand European only, 14.75% reported both New Zealand European and Māori ancestry, and 6.00% identified solely as Māori; one‐quarter identified with other ethnic groups. Most participants lived in urban settings, with nearly 60% residing in city suburbs. Reported household incomes covered a wide range, though a notable proportion fell below $75,000 or declined to disclose their income. Educational attainment was relatively high: over 40% held at least a bachelor's degree. Self‐assessed knowledge of inflation varied, with most respondents rating their understanding as moderate or good. Nearly all participants, 96.50%, reported having been involved in making household financial decisions in the preceding year.

**FIGURE 1:**
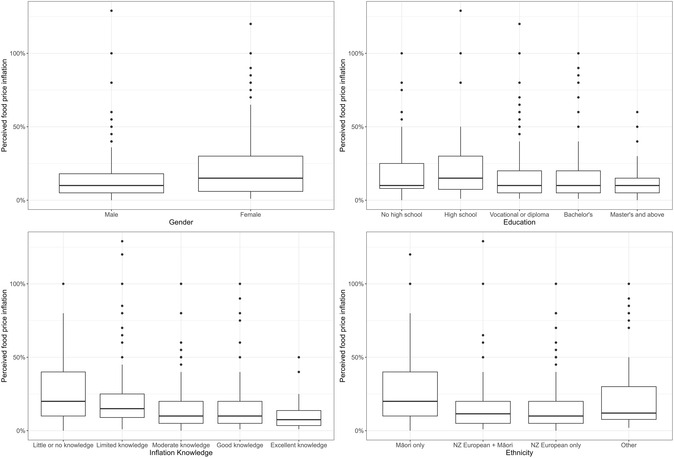
Distribution of food price perceptions across different segments.

The boxplots in Figure 1 illustrate the distribution of perceived food price inflation over the past 12 months, disaggregated by gender, education, inflation knowledge and ethnicity. All distributions are positively skewed with several outliers exceeding 75%. Females reported slightly higher perceived inflation than males, while Māori respondents reported systematically higher estimates, with both higher medians and greater dispersion relative to other ethnic groups. Perceived inflation declined with education and self‐reported inflation knowledge: respondents with tertiary qualifications or greater knowledge tended to report lower food price inflation. However, these differences are modest, with substantial overlaps in the interquartile ranges evident in each of the four panels.

Table 2 presents information on the prices that influenced people's perceptions of overall inflation, food prices that were most conspicuous, and how people managed increases in the cost of living. Grocery prices emerged as a key driver of perceived inflation, shaping both how respondents viewed price changes and how they adapted their household budgets. Interestingly, a considerably larger proportion of people reported grocery prices exerting a greater influence on their perceptions of inflation than petrol prices, which have traditionally attracted greater attention in both public discourse and academic research. This stands to reason as households purchase groceries more frequently than petrol and allocate a substantially larger share of their budgets to food.

**TABLE 2:** Perceived inflation, salient price categories and strategies to cope with inflation.

Question	Categories	Count	% of total
Changes in which expense category has had the biggest impact on your perception of inflation over the last 12 months?	Groceries	507	63.38%
	Mortgage	77	9.63%
	Petrol	68	8.50%
	Electricity	56	7.00%
	Eating out	32	4.00%
	Rent	27	3.38%
	Other	13	1.63%
	Healthcare	11	1.38%
	Gas	9	1.13%
Changes in the prices of which of the following do you notice the most?	Fresh fruits and vegetables	251	31.38%
	Unprocessed meats and eggs	184	23.00%
	Dairy	146	18.25%
	Restaurant meals	99	12.38%
	Packaged foods	82	10.25%
	Sweetened foods	20	2.50%
	Other	18	2.25%
What changes have you made to manage your cost of living?	Reduced non‐essential spending	555	69.38%
	Made different choices at the grocery store	414	51.75%
	Cut back on groceries or food expenses	368	46.00%
	Drove less	258	32.25%
	Delayed major purchases	247	30.88%
	Reduced energy consumption	244	30.50%
	Sought additional sources of income	91	11.38%
	Made no change	76	9.50%
	Bought an electric car	25	3.13%
	Put solar panels on my roof	24	3.00%
	Other	21	2.63%

More than half of the respondents reported altering their grocery purchases in response to rising living costs, and around 46.00% reduced their overall food spending. It is concerning that almost 80% of the respondents reported resorting to at least one of these two options to manage the effects of inflation. It suggests that inflationary pressure is not confined to discretionary spending but is reshaping essential consumption patterns in ways that may affect household welfare profoundly.

Price increases were most frequently noticed in fresh fruits and vegetables, unprocessed meats, and dairy. Just over 31% of the respondents reported that price changes in fresh fruits and vegetables were the most noticeable. Official data indicate that the prices of fruits and vegetables actually declined by 6.18% over the 12 months preceding the survey. Thus, it is particularly striking that respondents nonetheless perceived overall food prices to have risen by 18.14% during the same period. Furthermore, these items are generally associated with a healthy diet. As these prices rise and become more conspicuous, consumers may substitute away from them toward less nutritious, lower‐cost alternatives. This pattern aligns with recent evidence provided by Vatsa and Renwick (2025), showing a faster increase in the prices of healthy foods relative to unhealthy ones in New Zealand.

These findings suggest that food prices are not just shaping how people perceive inflation but may also influence what they eat. This makes it especially important to pay closer attention to people's perception of grocery prices when studying inflation and its effects on everyday life.

## 3 Methods

We first estimated a linear regression model to examine how demographic and behavioural characteristics were associated with perceived food price inflation. The model is specified as

(1) $$\pi_{i}^{p}=\alpha +\beta \prime X_{i}+\varepsilon_{i},$$

where $\pi_{i}^{p}$ denotes the food price inflation over the last 12 months reported by respondent ***i***, ***X* *_i_*** is a vector of control variables, ***β*** is the associated coefficient vector, and $\varepsilon_{i}$ is an independently and identically distributed error term. The model was estimated using ordinary least squares (OLS). While informative, this specification is limited in its ability to capture heterogeneity in the relationship between covariates and the outcome across the distribution of perceived food price inflation. With their focus exclusively on the conditional mean effect, the estimates are sensitive to outliers.

To address this, we estimated a quantile regression at the median of the conditional distribution of perceived food price inflation (Koenker 1994, 2010; Koenker and Bassett 1978)

(2) $$Q_{0.5}\left(\pi_{i}^{p}|X_{i}\right)=\alpha_{0.5}+\beta_{0.5}^{\prime }X_{i},$$

where $Q_{0.5}\;\;(\pi_{i}^{p}|X_{i})$ denotes the conditional median of perceived food price inflation, and $\beta_{0.5}$ captures the associations between the control variables and perceived food price inflation at the 50th percentile of its conditional distribution. Standard errors were obtained via nonparametric bootstrapping with 1,000 replications to account for heteroskedasticity and improve finite‐sample inference. The parameters were estimated by solving

(3) $$\min_{\alpha_{0.5},\beta_{0.5}}\sum_{i=1}^{n}\rho_{0.5}\left(\pi_{i}^{p}-\alpha_{0.5}-\beta_{0.5}^{\prime }X_{i}\right),$$

where $\rho_{0.5}(u)$ is the check function for the median; positive and negative residuals are penalised symmetrically.

We also estimated quantile regressions at the 25th and 75th percentiles to provide a more comprehensive characterisation of the heterogeneity in the associations beyond measures of central tendency. Specifically, $\rho_{0.25}(u)=u(0.25-I\{u\;<\;0\})$ and $\rho_{0.75}(u)=u(0.75-I\{u\;<\;0\})$ represent the check functions for the 25th and 75th percentiles, respectively. The asymmetric weights allow us to estimate how different parts of the outcome distribution respond, which is particularly useful considering the skewed distribution of reported food price inflation evident in Figure 1.

## 4 Results and Discussion

Table 3 presents the results from the regression described in Equation (1). Women reported significantly higher food price inflation than men, with a gap of 6.01 percentage points. While it may be tempting to attribute gender differences in perceived food price inflation to the assumption that women shop for groceries more frequently than men, this explanation is not supported by the data. We find no statistically significant difference in grocery shopping frequency between males and females, and our analysis explicitly controls for shopping frequency. The observed gender gap in inflation perceptions, therefore, reflects deeper behavioural or cognitive differences rather than differences in exposure.

**TABLE 3:** Linear regressions results.

Variable	Estimate	Std. Error
Intercept	31.93***	6.11
Ethnicity (Māori)		
European + Māori	−9.78*	4.40
European only	−11.27**	4.07
Other ethnicity	−4.57	4.25
Age	−0.10*	0.04
Location (City centre)		
City suburb	−0.24	2.63
Rural	2.57	2.94
Town	−1.82	4.34
Village	1.33	3.45
Gender (Male)		
Female	6.01***	1.46
Income ($0–24,999)		
$25,000–49,999	5.43	3.49
$50,000–74,999	−1.4	3.03
$75,000–99,999	2.41	3.19
$100,000–$124,999	−1.37	3.43
$125,000–$149,999	−4.02	3.10
$150,000–$174,999	−3.38	3.41
$175,000–$199,999	−2.68	4.04
$200,000 and above	−3.94	3.51
Undisclosed	−1.78	3.26
Education (No or low education)		
High‐school diploma	1.85	2.58
Vocational or technical diploma	−0.93	2.54
Bachelor's degree	−2.66	2.39
Master's degree or above	−4.73^	2.48
Inflation knowledge (Little or no knowledge)		
Limited knowledge	−1.72	4.41
Moderate knowledge	−4.37	4.31
Good knowledge	−3.84	4.49
Extensive knowledge	−3.2	5.32
Grocery purchases per month	0.09	0.16

*Notes:* ***, **, *, and ^ denote statistical significance at the 0.1%, 1%, 5%, and 10% significance levels, respectively. Reference categories are in parentheses. Heteroscedasticity‐robust standard errors (Std. Error) are reported.

Ethnic disparities were also notable. Compared to Māori respondents, those identifying as both Māori and European reported food price inflation perceptions that were 9.78 percentage points lower; this figure was 11.27 percentage points for those identifying as European only. Older individuals reported lower inflation, with a marginal effect of –0.10 per year of age. Among education categories, only those with at least a master's degree differed at the 10% significance level from respondents in the reference group, i.e., those without formal education, estimating inflation to be 4.73 percentage points lower. No significant differences were observed in terms of location, income, or self‐assessed knowledge of inflation.

**FIGURE 2:**
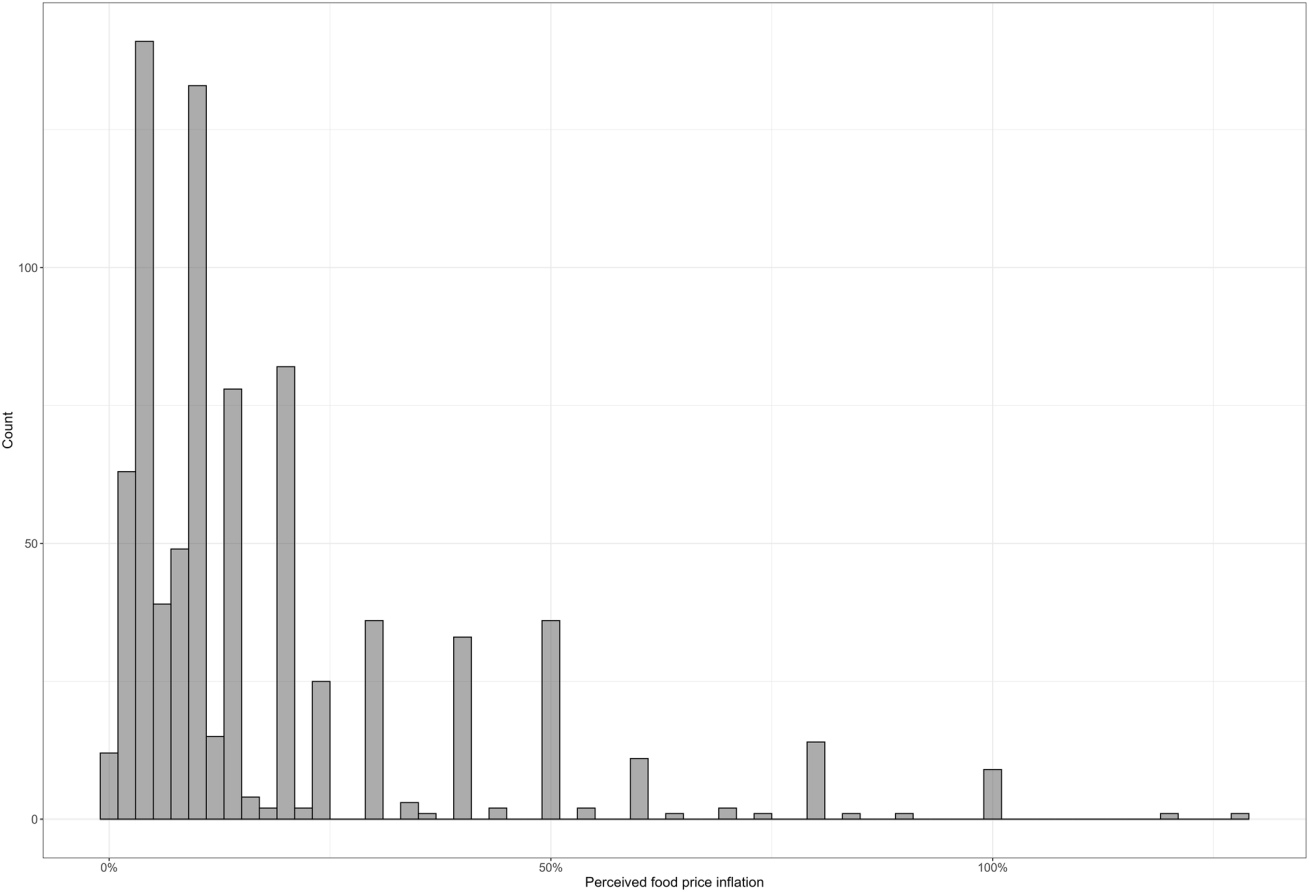
Distribution of perceptions of food price inflation.

As shown in Figure 2, the distribution of reported food price inflation over the previous 12 months is right‐skewed, with most respondents concentrated below 30% while a considerable number reported much higher values. These outliers raise the mean and may bias OLS estimates. Considering this, it is informative to examine how different variables were associated with perceptions of food price inflation at various points in the distribution, not just at the mean. To this end, we estimated quantile regressions at the 25th, 50th and 75th percentiles.

The results obtained from these regressions are reported in Table 4. They are noticeably different on several counts. At the median, our results show no statistically significant differences in the perceptions of food price inflation among people of different ethnicities. Age and gender, however, were associated with differences in these perceptions. The coefficient of age, –0.08, was significant at the 5% significance level and close to the corresponding estimate obtained from the linear regression. The coefficient of female was noticeably smaller, though. At the median, females perceived inflation to be 3.39 percentage points higher than males.

**TABLE 4:** Quantile regression results.

	*τ* = 0.25		*τ* = 0.50			*τ* = 0.75		
Variable	Estimate	Std. Error	Estimate	Std. Error		Estimate	Std. Error	
Intercept	10.75**	3.31	22.37***	6.39	44.01***			12.72
Ethnicity (Māori)								
European + Māori	−2.11	2.41	−5.28	4.08	−9.49			8.19
European only	−2.45	2.24	−6.45^	3.79	−14.83^			7.85
Other ethnicity	−0.86	2.32	−4.11	4.01	−7.10			8.47
Age	−0.01	0.02	−0.08*	0.03	−0.16*			0.07
Location (City centre)								
City suburb	−0.5	1.50	−0.21	2.25	−0.64			3.36
Rural	0.12	1.71	1.94	2.40	3.39			3.85
Town	−0.55	1.91	−1.49	2.98	−2.29			7.70
Village	−1.23	1.81	−0.15	2.73	6.37			6.11
Gender (Male)								
Female	0.82	0.57	3.39**	1.08	7.95***			2.06
Income ($0–24,999)								
$25,000–49,999	0.47	1.36	2.30	3.44	1.95			6.43
$50,000–74,999	−0.39	1.27	−0.53	3.39	−4.89			5.99
$75,000–99,999	1.62	1.33	2.03	3.44	1.52			6.35
$100,000–$124,999	−1.26	1.42	−0.84	3.55	−3.76			6.14
$125,000–$149,999	−0.24	1.62	−0.70	3.52	−5.91			6.25
$150,000–$174,999	0.18	1.57	0.09	3.62	−6.44			6.02
$175,000–$199,999	−1.35	1.81	−1.18	3.66	−6.94			6.27
$200,000 and above	−0.92	1.40	−1.09	3.37	−7.30			5.82
Undisclosed	−1.57	1.28	−1.37	3.48	−4.71			6.49
Education (No or low education)								
High‐school diploma	0.38	0.99	3.55	2.05	4.97			5.20
Vocational or technical diploma	−0.97	0.96	0.14	1.61	0.40			4.95
Bachelor's degree	−0.99	0.95	0.26	1.69	−2.17			4.84
Master's degree or above	−0.42	0.95	−0.46	1.78	−0.99			5.10
Inflation knowledge (Little or no knowledge)								
Limited knowledge	0.57	2.48	−3.22	4.16	−2.77			9.15
Moderate knowledge	−1.36	2.51	−4.66	4.11	−5.97			8.91
Good knowledge	−1.04	2.53	−4.30	4.23	−5.59			9.03
Extensive knowledge	−1.44	2.79	−4.59	4.81	−4.38			10.65
Frequency of monthly grocery purchases	−0.08	0.05	−0.07	0.08	0.01			0.20

*Notes:* ***, **, *, and ^ denote statistical significance at the 0.1%, 1%, 5%, and 10% significance levels, respectively. Reference categories are in parentheses. Standard errors (Std. Error) are bootstrapped using 1,000 replications.

None of the variables was statistically significant at the 25th percentile of the distribution. This suggests that among respondents reporting relatively low food price inflation, demographic and socioeconomic characteristics such as gender, ethnicity, education and income offered little explanatory power. These individuals may have more accurate information and more stable consumption patterns, resulting in perceptions that are less variable. It bears emphasising, however, that perceived food price inflation at the 25th percentile was 5.00%, more than 2 percentage points higher than the rate reported in official statistics. In conjunction with the absence of systematic differences across demographic and behavioural characteristics, this points to a broadly shared view that diverged markedly from official estimates.

At the 75th percentile, the coefficients of age and gender were considerably larger than those at the median and the ones derived from the linear regression, suggesting that the factors that drive high inflation perceptions appear to matter more in the upper tail. Even though the coefficients were larger in magnitude, their signs remained consistent across different specifications and percentiles of the distribution. The coefficient of age was consistently negative, whereas that of gender was positive.

Individuals who believe inflation to be high, regardless of the official rate, may curtail spending, which in turn could dampen economic activity. But the importance of food transcends economic policy. It has important implications for food security, diet, and health. Vatsa and Renwick (2025) showed that in the decade leading up to 2023, prices of fresh fruits and vegetables in New Zealand increased more rapidly than those of processed, sweetened foods. This relative price shift may encourage consumers to substitute healthier options with cheaper, less nutritious alternatives, potentially leading to poorer dietary quality and long‐term health challenges. Such changes in consumption patterns could, over time, impose greater costs and strain on the health system. Our data show that individuals who perceived food price inflation to be less than 5% were significantly less likely to reduce grocery spending or alter their choices at the grocery store compared to those who perceived food price inflation to be higher than 25%. This highlights the importance of ensuring that inflation perceptions are aligned with actual price movements—misperceptions can lead people to unnecessarily compromise on food quality, with potential consequences for health and wellbeing.

## 5 Concluding Remarks

Food prices rose sharply in New Zealand between June 2021 and June 2023, with food price inflation increasing from 2.8% to 12.5%; but by June 2024, food price inflation had fallen to –0.3%. Nevertheless, the prolonged exposure to rapidly rising prices may have distorted households’ perceptions, prompting changes in spending behaviour and dietary choices, including the substitution of healthier foods with cheaper, less nutritious alternatives.

This paper investigates how New Zealand households perceive food price inflation, how these perceptions vary across socio‐demographic groups, and whether coping with rising prices involves buying and eating differently. Using novel household survey data collected in February 2025, we find that perceived food price inflation was substantially higher than the official rate. At the time of the survey, annual food price inflation stood at approximately 2.3%, yet households believed it to be 18.1%, which is more than seven times greater. Females reported higher inflation than males, and perceptions declined with age. These differences were concentrated in the middle and upper parts of the distribution. While mean‐based estimates suggested notable ethnic differences, particularly between Māori and New Zealanders of European descent, results from quantile regressions did not provide strong support for these patterns.

Overall, our findings suggest that New Zealanders, regardless of income, gender, ethnicity, education, or self‐assessed inflation knowledge, perceived food price inflation to be significantly higher than official statistics indicate. This misalignment is concerning, as it may influence what people eat and how much they spend on food, with potential consequences for health and wellbeing. It cannot be ruled out that official statistics, which underpin economic policy, do not fully reflect the lived experience of households (D’Acunto et al. 2023). Consumers may accurately perceive changes in their own food expenses yet misjudge the overall rate of food price inflation. It is also possible that experiencing a cost‐of‐living crisis may have left a lasting, albeit inaccurate, impression that food price inflation remains elevated. Many may be conflating high food prices with rapidly rising food prices.

We hope the findings in this article will stimulate further research into the drivers of this misalignment and inform efforts to bridge the gap between statistical reporting and public perception. Improving communication around food price trends may help households make better‐informed choices and reduce unintended consequences for nutrition and health. We have not modelled uncertainty in perceptions of food price inflation. Future surveys should capture this uncertainty to enable analysis of higher moments of the distribution of these perceptions and offer a more nuanced understanding of how individuals perceive changes in food prices.

## Funding

This work was supported by the Lincoln University Faculty of Agribusiness and Commerce Seed Funds and Lincoln University Centre of Excellence in Transformative Agribusiness.

## Conflicts of Interest

The authors declare no conflicts of interest.

## References

[snz270018-bib-0001] Adjemian, M. K. , S. Arita , S. Meyer , and D. Salin . 2024. “Factors Affecting Recent Food Price Inflation in the United States.” Applied Economic Perspectives and Policy 46, no. 2: 648–676. 10.1002/aepp.13378.

[snz270018-bib-0002] Anesti, N. , V. Esady , and M. Naylor . 2025. “Food Prices Matter Most: Sensitive Household Inflation Expectations.” (Bank of England Staff Working Paper No. 1125).

[snz270018-bib-0003] Binder, C. C. 2018. “Inflation Expectations and the Price at the Pump.” Journal of Macroeconomics 58: 1–18. 10.1016/j.jmacro.2018.08.006.

[snz270018-bib-0004] Binder, C. C. , and C. Makridis . 2022. “Stuck in the Seventies: Gas Prices and Consumer Sentiment.” Review of Economics and Statistics 104, no. 2: 293–305. 10.1162/rest_a_00944.

[snz270018-bib-0005] Coibion, O. , and Y. Gorodnichenko . 2015. “Is the Phillips Curve Alive and Well after All? Inflation Expectations and the Missing Disinflation.” American Economic Journal: Macroeconomics 7, no. 1: 197–232. 10.1257/mac.20130306.

[snz270018-bib-0006] D’Acunto, F. , U. Malmendier , and M. Weber . 2023. “What Do the Data Tell Us about Inflation Expectations?” In Handbook of Economic Expectations. 133–161. Elsevier Inc. 10.1016/B978-0-12-822927-9.00012-4.

[snz270018-bib-0007] FAO , IFAD , UNICEF , WFP , WHO . 2025. The State of Food Security and Nutrition in the World 2025 – Addressing High Food Price Inflation for Food Security and Nutrition. FAO. IFAD, UNICEF, WFP, WHO. 10.4060/cd6008en.

[snz270018-bib-0008] Jonung, L. 1981. “Perceived and Expected Rates of Inflation in Sweden.” American Economic Review 71, no. 5: 961–968.

[snz270018-bib-0009] Kikuchi, J. , and Y. Nakazono . 2023. “The Formation of Inflation Expectations: Microdata Evidence from Japan.” Journal of Money, Credit and Banking 55, no. 6: 1609–1632. 10.1111/jmcb.12944.

[snz270018-bib-0010] Kilian, L. , and X. Zhou . 2022a. “Oil pPrices, Gasoline Prices, and Inflation Expectations.” Journal of Applied Econometrics 37, no. 5: 867–881. 10.1002/jae.2911.

[snz270018-bib-0011] Kilian, L. , and X. Zhou . 2022b. “The Impact of Rising Oil Prices on U.S. Inflation and Inflation Expectations in 2020‐23.” Energy Economics 113: 106228. 10.1016/j.eneco.2022.106228.

[snz270018-bib-0012] Kilian, L. , and X. Zhou . 2025. “Oil Price Shocks and Inflation.” In Research Handbook of Inflation. Edited by G. Ascari and R. Trezzi , Edward Elgar Publishing Ltd.

[snz270018-bib-0013] Koenker, R. 1994. “Confidence Intervals for Regression Quantiles.” In Asymptotic Statistics. Contributions to Statistics. Edited by P. Mandl , M. Hušková . Physica. 10.1007/978-3-642-57984-4_29.

[snz270018-bib-0014] Koenker, R. 2010. “Quantile Regression.” In Quantile Regression. Cambridge University Press. 10.1017/CBO9780511754098.

[snz270018-bib-0015] Koenker, R. , and G. Bassett Jr . 1978. “Regression Quantiles.” Econometrica 46, no. 1: 33–50.

[snz270018-bib-0016] Tversky, A. , and D. Kahneman . 1974. “Judgment under Uncertainty: Heuristics and Biases.” Science 185, no. 4157: 1124–1131. 10.1126/science.185.4157.1124. 17835457

[snz270018-bib-0017] Vatsa, P. 2025. “Oil and Petrol Prices, Inflation Perceptions, and Inflation Expectations: Evidence from New Zealand.” Applied Economics 1–12.

[snz270018-bib-0018] Vatsa, P. , and G. Pino . 2024. “Do Petrol Prices Affect Inflation and Inflation Expectations? Evidence from New Zealand.” Energy Economics 139: 107939. 10.1016/j.eneco.2024.107939.

[snz270018-bib-0019] Vatsa, P. , and G. Pino . 2025a. “Inflation Perceptions and Petrol Prices in New Zealand.” New Zealand Economic Papers 1–11. 10.1080/00779954.2025.2562988.

[snz270018-bib-0020] Vatsa, P. , and G. Pino . 2025b. “Petrol Prices, Food Prices, and Inflation Perceptions in New Zealand—Evidence From a Threshold Analysis.” Australian Economic Papers 64, no. 2: 170–178. 10.1111/1467-8454.12388.

[snz270018-bib-0021] Vatsa, P. , and A. Renwick . 2025. “Food Prices in New Zealand: Implications for Feeding People Better.” Journal of the Royal Society of New Zealand 55, no. 6: 2305–2318. 10.1080/03036758.2024.2368788. 40756862 PMC12315146

[snz270018-bib-0022] Weber, M. , Y. Gorodnichenko , and O. Coibion . 2022. “The Expected, Perceived, and Realized Inflation of U.S. Households before and during the COVID19 Pandemic.” National Bureau of Economic Research, Working Paper No. 29640. 10.3386/w29640.

